# Synchronous splenectomy and hepatectomy for patients with small hepatocellular carcinoma and pathological spleen: neutrophil to lymphocyte ratio changes can predict the prognosis

**DOI:** 10.18632/oncotarget.17758

**Published:** 2017-05-10

**Authors:** Xiaoyun Zhang, Chuan Li, Tianfu Wen, Wei Peng, Lunan Yan, Bo Li, Jiayin Yang, Wentao Wang, Mingqing Xu, Yong Zeng

**Affiliations:** ^1^ Department of Liver Surgery and Liver Transplantation Center, West China Hospital of Sichuan University, Chengdu 610041, Sichuan Province, China

**Keywords:** hepatocellular carcinoma, splenomegaly, hypersplenism, hepatectomy, neutrophil to lymphocyte ratio

## Abstract

**Background:**

Treatments about small hepatocellular carcinoma (HCC) and hypersplenism associated with good hepatic reserve are not well established. The aim of this study was to investigate the outcome of synchronous hepatectomy and splenectomy for those patients.

**Study Design:**

Splenomegaly and hypersplenism were defined as a pathological spleen. Seven hundred fifty-six patients with small HCC (381 with a pathological spleen, 375 without a pathological spleen) were divided into three groups. One hundred ten of 381 patients underwent synchronous hepatectomy and splenectomy (group A), 271 of 381 patients underwent hepatectomy alone (Group B) and 375 patients without pathological spleen underwent hepatectomy alone (Group C).

The influence of pathological spleen, outcome of different treatments and systemic inflammatory response indexes were analyzed.

**Results:**

Both overall survival (OS, P=0.023) and disease-free survival (DFS, P=0.020) were significantly increased in group C compared to group B. A pathological spleen was a significant independent prognostic factor for OS and DFS among those two groups. In addition, OS (P=0.025) and DFS (P=0.004) were increased in the group A compared to group B. Splenectomy and neutrophil to lymphocyte ratio changes (ΔNLR) were significant independent prognostic factors of the prognosis for patients in groups A and B.

**Conclusions:**

A Pathological spleen influences the outcome of HCC patients. Synchronous hepatectomy and splenectomy should be performed among patients with small HCC and a pathological spleen. ΔNLR can predict the prognosis of these patients.

## INTRODUCTION

Cirrhotic hypersplenism secondary to portal hypertension is commonly associated with hepatocellular carcinoma (HCC), resulting in anemia, leucopenia, and thrombocytopenia [1–3]. HCC patients with advanced liver disease (significant portal hypertension, elevated bilirubin, etc) may be better served by liver transplantation [4]. However, the treatment is not well established among HCC patients with hypersplenism and good hepatic reserve. Recently, synchronous hepatectomy and splenectomy have been applied as another option to treat HCC and cirrhotic hypersplenism [5–8] especially in Japan and China. Unfortunately, many of these trials lacked adequate sample sizes and long-term follow-up and were heterogeneous with respect to the patient populations, thereby limiting their ability to produce robust conclusions.

In addition, increasing evidence suggests a correlation between the presence of systemic inflammation and poor outcomes of HCC [9–11]. Through the promotion of angiogenesis, damage to DNA, and inhibition of apoptosis, the systemic inflammatory response results in upregulation cytokines and inflammatory mediators and predisposes the tumor to proliferate and metastasize [12, 13]. Various systemic inflammatory response indexes, such as, absolute blood neutrophil or lymphocyte counts, the neutrophil to lymphocyte ratio (NLR), the platelet to lymphocyte ratio (PLR), and the change in neutrophil to lymphocyte ratio (ΔNLR) can be used to evaluate systemic inflammation [10, 14, 15]. An inflammation-based index that is more suitable for predicting the outcome of patients with HCC and hypersplenism has not been established.

Thus, this study was designed to examine the outcome of HCC patients (within Milan Criteria) with hypersplenism following synchronous hepatectomy and splenectomy with a focus on changes in systemic inflammation.

## RESULTS

### Splenomegaly and hypersplenism affect the outcome of HCC patients after surgical resection

All patients in groups B and group C underwent hepatectomy alone. Patients in group B exhibited a pathological spleen, whereas patients in group C did not have this condition. Therefore, we explored the influence of pathological spleen among those two groups.

The baseline characteristics between groups B and C were comparable except pathological for spleen [number with splenomegaly (P<0.001), white blood cell (WBC) count (P=0.047), platelet (PLT) count (P<0.001), diameter of tumor (P=0.012), micro-vascular invasion (P=0.043) and cirrhosis (P=0.001)] (Table 1).

**Table 1:** Baseline characteristics of group A, group B and group C

Variables	Group A	Group B	P *	Group C	P ^†^
Number of patients (n)	110	271		375	
Age (years)	50.19±9.74	49.99±14.23	0.027	49.39±13.98	0.911
Gender (male/female)	91:19	229:42	0.647	323:52	0.573
HBsAg (positive:negative)	103:7	253:18	1.000	347:28	0.758
AFP (≤400: >400, ng/ml)	77:33	193:78	0.805	262:113	0.727
TBIL(umol/L)	17.84±6.66	16.61±6.78	0.586	14.72±11.82	0.861
AST (IU/L)	40.85±19.95	43.13±26.21	0.206	38.37±42.49	0.732
ALB (g/L)	39.94±6.04	41.54±4.64	0.139	41.54±4.64	0.625
PT (s)	13.38±1.43	12.83±4.65	0.453	12.37±1.05	0.349
Pathological spleen					
Splenomegaly(I°/II°/III°), n	16:72:22	226:39:6	<0.001	-	-
WBC (10^9^/L)	3.01±1.48	4.53±1.66	0.286	5.79±1.80	0.047
PLT (10^9^/L)	53.48±35.63	75.34±18.69	<0.001	150.35±46.59	<0.001
Number of tumors (single:multiple)	100:10	239:32	0.588	346:29	0.101
Diameter of tumor (cm)					
Mean	3.32±1.12	3.14±1.05	0.131	3.34±1.12	0.012
≤3	61 (55.5%)	151 (55.7%)	1.000	183 (48.8%)	0.094
3-5	49 (44.5%)	120 (45.3%)		192 (51.2%)	
MVI (yes:no)	15:95	55:216	0.146	53:322	0.043
Differentiation, n (%)					
High	6 (5.5%)	12 (4.4%)	0.125	19 (5.1%)	0.098
Moderate	72 (65.5%)	150 (55.4%)		236 (62.9%)	
Low	32 (29.0%)	109 (40.2%)		120 (32.0%)	
Cirrhosis (yes vs. no)	105:5	252:19	0.487	288:87	0.001

HBsAg: hepatitis b surface antigen; AFP: alpha-fetoprotein; TBIL: total bilirubin; AST: aspartate aminotransferase; ALB: serum albumin; PLT: platelet; WBC: white blood cell; MVI: Micro-vascular invasion; grade I: spleen enlarging beyond left subcostal margin and palpable; grade II: spleen reaching umbilicus; grade III: spleen extending into pelvic cavity; Cirrhosis: yes: Ishak ≥5; no: Ishak <5

*Indicates that group B compares to group A; ^†^ indicates that group B compares to group C.

Regarding short-term outcomes, HCC patients with a pathological spleen in group B underwent more transfusion and had a longer hospital stay than patients in group C (transfusion: 11.1% vs. 3.0%, P<0.001; hospital stay: 8.03±4.72 days vs. 7.51±2.83 days, P=0.006) (Table 2).

**Table 2:** Major complication classification and other clinical data of the three groups

Variables	GroupA(n=110)	GroupB(n=271)	P*	GroupC(n=375)	P^†^
Transfusion, yes vs. no	12:98	27:244 (11.1%)	0.852	11:364 (3.0%)	<0.001
Hospital stays (days)	8.46±3.41	8.03±4.72	0.671	7.51±2.83	0.006
Type of Resection^‡^ (major/minor)	11:99	44:227	0.147	71:304	0.405
Intraoperative bleeding (mL) mean	378.90±401.11	361.00±292.88	0.223	342.54±344.71	0.343
No. of death, n (%)	25(22.7%)	81(29.9%)	0.167	69(18.4%)	0.001
No. of recurrence, n (%)	37(33.6%)	129(47.6%)	0.016	130(34.7%)	0.001
Mean time to recurrence (months)	36.26±25.77	25.38±22.45	<0.001	27.05±23.68	0.366
Mean follow-up time (months)	41.69±25.12	35.51±24.06	0.025	34.23±26.42	0.527
Clavien classification, n	110	273		377	
Grade IIIa^§^	1(0.9%)	4(1.5%)		5(1.3%)	
IIIb^||^	3(2.7%)	2(0.7%)		2(0.5%)	
Grade IV^¶^	1(0.9%)	1(0.4%)		2(0.5%)	
Grade V^#^	1(0.9%)	2(0.7%)		2(0.5%)	
Total	6(5.4%)	9(3.3%)	0.384	11(2.8%)	0.820

*Indicates that group B compares to group A; ^†^indicates that group B compares to group C.

^‡^Major resection = resection of three or more segments.

^§^Group A: 1 patient received abdominocentesis; group B: 3 patient received abdominocentesis and 1 underwent pleurocentesis; group C: 1 patient had abdominocentesis and 4 had pleurocentesis.

^||^Group A: 3 patients underwent re-laparotomy for intra-abdominal bleeding; group B: 2 patients underwent re-laparotomy for intra-abdominal bleeding; group C: 1 patient underwent re-laparotomy for intra-abdominal bleeding; 1 patient received incision and drainage for abdominal abscess.

^¶^Group A: 1 patients needed ICU management due to the hypotension and shock; group B: 1 patient suffered type I respiratory; group C: 2 patients suffered type I respiratory.

^#^Group A: 1 patient died of liver failure; group B: 1 patient died of liver failure and 1 died of intra-abdominal bleeding post-discharge within three months; group C: 1 patient died of liver failure and 1 died of intra-abdominal bleeding post-discharge within three months.

Regarding long-term outcomes, increased recurrence and death were noted in group B compared to group C (recurrence: 47.6% vs. 34.7%, P=0.001; death: 29.9% vs. 18.4%, P=0.001) (Table 2). HCC patients in group C exhibited a significantly better overall survival rate than patients in group B (the 1-, 3-, 5-, 7-, and 9-year overall survival rates: 95.6%, 79.0%, 71.9%, 60.7%, and 60.7%, respectively VS. 93.5%, 75.7%, 56.3%, 40.0%, and 27.8%, respectively, P=0.023)(Figure 1A). Disease-free survival rates in group C were also significantly increased compared to group B (1-, 3-, 5-, 7-, and 9-year overall survival rates: 82.6%, 64.9%, 49.7%, 35.4%, and 27.2%, respectively vs. 71.9%, 50.6%, 40.6%, 27.0%, and 21.6%, respectively, P=0.020)(Figure 1B).

**Figure 1A:**
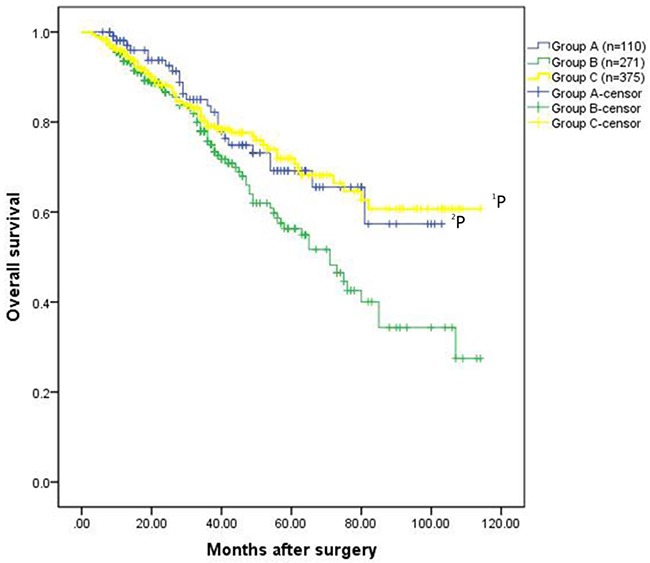
Overall survival rates for the patients in the three groups The patients exhibited HCC without hypersplenism in the group C had much better overall survival rate than the patients with HCC and hypersplenism in the group B (the 1-, 3-, 5-, 7-, and 9-year overall survival rates: 95.6%, 79.0%, 71.9%, 60.7%, and 60.7%, respectively VS. 93.5%, 75.7%, 56.3%, 40.0%, and 27.8%, respectively,^1^P=0.023). The overall survival rate for patients underwent hepatectomy and spleencetomy in the group A was significantly better than that of patients in the group B (the 1-, 3-, 5-, 7-, and 9-year overall survival rates: 98.1%, 83.6%, 69.2%, 57.3%, and 57.3%, respectively VS. 93.5%, 75.7%, 56.3%, 40.0%, and 27.8%, respectively, ^2^P=0.025).

**Figure 1B:**
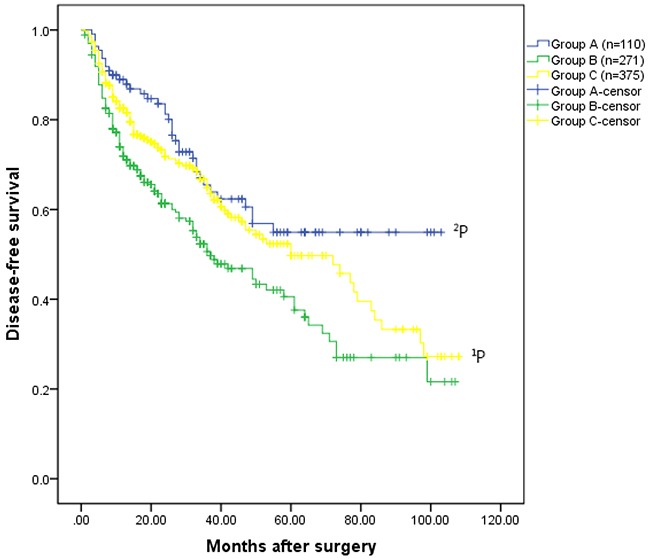
Disease-free survival rates for the three groups The patients had HCC without hypersplenism in the group C had much better disease-free survival rate than the patients with HCC and hypersplenism in the group B (the 1-, 3-, 5-, 7-, and 9-year overall survival rates: 82.6%, 64.9%, 49.7%, 35.4%, and 27.2%, respectively VS. 71.9%, 50.6%, 40.6%, 27.0%, and 21.6%, respectively, ^1^P =0.020). The disease-free survival rate for patients underwent hepatectomy and spleencetomy in the group A was significantly better than that of patients in the group B (the 1-, 3-, 5-, 7-, and 9-year disease-free survival rates: 89.0%, 65.5%, 54.9%, 54.9%, and 54.9%, respectively VS. 71.9%, 50.6%, 40.6%, 27.0%, and 21.6%, respectively,^2^P =0.004).

Further univariate and multivariate analyses using the Cox proportional hazard model identified that pathological spleen (HR=1.485, 95%CI: 1.075-2.052, P=0.016) and MVI (HR=1.708, 95%CI: 1.190-2.451, P=0.004) were significant independent prognostic factors for overall survival among HCC patients who underwent hepatectomy alone (group B and group C, n=646) (Table 3). In addition, the univariate analysis indicated that two variables were statistically significant prognostic factors associated with disease-free survival in these patients: the presence of pathological spleen (P = 0.018), and MVI (P = 0.032). Furthermore, based on multivariate analysis, pathological spleen (HR=1.390, 95%CI: 1.087-1.778, P=0.009), degree of differentiation (HR = 1.283; 95%CI: 1.029-1.599; P=0.027) and MVI (HR=1.408, 95%CI: 1.050-1.890, P=0.022) were independent prognostic factors for disease-free survival (Table 3).

**Table 3:** Univariate and multivariate analyses of prognostic factors for overall survival and disease-free survival in patients underwent hepatectomy alone (n=646)

Variables	Overall survival				Disease-free survival			
	Univariate analysis		Multivariate analysis		Univariate analysis		Multivariate analysis	
	HR (95%CI)	P value	HR (95%CI)	P value	HR (95%CI)	P value	HR (95%CI)	P value
Gender, (F vs. M)	0.988(0.609-1.605)	0.962			1.023(0.717-1.460)	0.899		
Age, (≥60 vs. <60 y)	0.860(0.576-1.284)	0.462			0.831(0.618-1.119)	0.223		
HBsAg, (positive vs. negative)	1.334(0.631-2.821)	0.451			1.085(0.663-1.774)	0.746		
AFP, (≥400 vs.<400 ng/ml	1.388(0.980-1.966)	0.065			1.208(0.922-1.584)	0.170		
TBIL	1.005(0.991-1.019)	0.459			1.009(0.997-1.021)	0.149		
AST	0.998(0.993-1.068)	0.583			1.000(0.997-1.004)	0.848		
ALB	1.029(0.991-1.068)	0.135			1.014(0.985-1.044)	0.345		
Type of Resection (Major vs. Minor)	1.506(0.979-2.316)	0.062			1.116(0.793-1.569)	0.530		
Transfusion (yes vs. no)	0.789(0.432-1.441)	0.441			0.864(0.530-1.406)	0.556		
Pathological spleen, (yes vs. no)	1.470(1.050-2.057)	0.025	1.485(1.075-2.052)	0.016	1.368(1.055-1.774)	0.018	1.390 (1.087-1.778)	0.009
Differentiation (W, M, L)	1.188(0.871-1.621)	0.277			1.253(0.995-1.578)	0.055	1.283 (1.029-1.599)	0.027
Tumor number (single vs. multiple)	0.896(0.497-1.618)	0.717			1.292(0.832-2.005)	0.253		
Tumor size, (3- 5 vs. <3cm)	0.869(0.972-0.697)	0.869			0.998(0.766-1.275)	0.928		
MVI (yes vs. no)	1.663(1.146-2.414)	0.007	1.708(1.190-2.451)	0.004	1.398(1.030-1.898)	0.032	1.408 (1.050-1.890)	0.022
Cirrhosis (yes vs. no)	1.271(0.719-2.246)	0.410			1.246(0.834-1.861)	0.282		

Thus, we concluded that a pathological spleen influences the short-term and long-term outcomes of HCC patients after surgical resection. The presence of pathological spleen prolonged the hospital stay and increased transfusion rates. Moreover, a pathological spleen was a significant risk factor for tumor recurrence and long-term survival.

### HCC patients with pathological spleen benefit from synchronous hepatectomy and splenectomy

Patients in groups A and B exhibited a pathological spleen. Patients in group A underwent synchronous hepatectomy and splenectomy, whereas patients in group B underwent hepatectomy alone. Thus, we compared the outcome of between these two groups.

Table 1 also presents the baseline characteristics between groups A and B. No significant differences were noted for gender, hepatitis b surface antigen (HBsAg), alpha-fetoprotein (AFP), WBC count, number of tumors, maximum diameter, MVI, degree of differentiation and cirrhosis. However, patients in group A were older (50.19±9.74 vs. 49.99±14.23, P=0.027) and PLT count (53.48±35.63 vs. 75.34±18.69, P<0.001) was reduced compared to group B. The number of transfusions, length of hospital stay, type of resection, intraoperative bleeding and major complications were similarly between the two groups (Table 2). However, both the mean time to recurrence (36.26±25.77 vs. 25.38±22.45, P<0.001) and follow-up time (41.69±25.12 vs. 35.51±24.06, P=0.005) in group A were increased compared to group B.

Regarding long-term outcomes, the overall survival rate for patients who underwent hepatectomy and splenectomy in group A was significantly greater than those in group B (1-, 3-, 5-, 7-, and 9-year overall survival rates: 98.1%, 83.6%, 69.2%, 57.3%, and 57.3%, respectively vs. 93.5%, 75.7%, 56.3%, 40.0%, and 27.8%, respectively, P=0.025). Similarly, the 1-, 3-, 5-, 7-, and 9-year disease-free survival rates in group A were 89.0%, 65.5%, 54.9%, 54.9%, and 54.9%, respectively, and were significantly increased compared to group B (71.9%, 50.6%, 40.6%, 27.0%, and 21.6%, respectively, P=0.004). Although overall survival, not disease-free survival, of patients in the group A was comparable to that of patients underwent hepatectomy alone group (group B and group C, Supplementary Figure 1A and 1B).

Cox proportional hazard model revealed that MVI (HR=1.891, 95%CI: 1.183-3.022, P=0.008) was a significant independent prognostic factors for disease-free survival among HCC patients with pathological spleen (Table 4).

**Table 4:** Univariate and multivariate analyses of prognostic factors for overall survival and disease-free survival in HCC patients with pathological spleen (n=381)

Variables	Overall survival				Disease-free survival			
	Univariate analysis		Multivariate analysis		Univariate analysis		Multivariate analysis	
	HR (95%CI)	P value	HR (95%CI)	P value	HR (95%CI)	P value	HR (95%CI)	P value
Gender, (F vs. M)	1.256(0.775-2.037)	0.354			1.112(0.601-2.055)	0.736		
Age, (≥60 vs. <60 y)	0.727(0.481-1.097)	0.128			0.844(0.502-1.418)	0.522		
HBsAg, (positive vs. negative)	0.667(0.354-1.258)	0.211			0.717(0.293-1.752)	0.465		
AFP, (≥400 vs.<400 ng/mL	1.430(1.015-2.016)	0.041	1.459(1.055-2.016)	0.022	1.434(0.921-2.231)	0.110		
TBIL	1.013(0.989-1.036)	0.294			1.015(0.987-1.043)	0.305		
AST	0.999(0.992-1.005)	0.727			0.993(0.984-1.003)	0.159		
ALB	1.004(0.973-1.037)	0.795			0.997(0.958-1.038)	0.887		
Type of resection (major vs. minor)	1.364(0.872-2.134)	0.174			0.980(0.535-1.793)	0.947		
Transfusion (yes vs. no)	1.133(0.690-1.858)	0.622			0.964(0.524-1.774)	0.907		
Splenectomy, (yes vs. no)	0.555(0.358-0.860)	0.008	0.552(0.381-0.799)	0.002	0.567(0.326-1.018)	0.058	0.652 (0.414-1.028)	0.066
Preoperative-PLT	0.998(0.986-1.009)	0.687			0.997(0.983-1.012)	0.717		
Preoperative -WBC	0.857(0.541-1.358)	0.511			0.908(0.542-1.522)	0.714		
Preoperative - neutrophil	1.356(0.761-2.416)	0.301			1.439(0.709-2.922)	0.313		
Preoperative - lymphocyte	0.940(0.456-1.939)	0.868			0.588(0.218-1.585)	0.294		
Preoperative -NLR	0.931(0.787-1.103)	0.409			0.908(0.712-1.159)	0.439		
Preoperative -PLR	1.002(0.994-1.011)	0.603			1.000(0.989-1.011)	0.976		
Differentiation (W, M, L)	1.197(0.895-1.600)	0.225			1.010(0.699-1.458)	0.959		
Tumor number (single vs. multiple)	0.819(0.418-1.602)	0.559			1.222(0.557-2.685)	0.617		
Tumor size, (3- 5 vs. <3cm)	0.980(0.701-1.370)	0.904			1.090(0.712-1.667)	0.693		
MVI (yes vs. no)	1.428(0.973-2.096)	0.069	1.485(1.040-2.121)	0.029	1.891(1.183-3.022)	0.008	1.668 (1.082-2.571)	0.021
Cirrhosis (yes vs. no)	0.794(0.418-1.510)	0.482			0.975(0.407-2.338)	0.955		

In addition, the univariate analysis indicated that two variables were statistically significant prognostic factors associated with overall survival in HCC patients with pathological spleen: AFP level (P = 0.041), and splenectomy (P = 0.008). Furthermore, based on the multivariate analysis, AFP level (HR=1.459, 95%CI: 1.055-2.016, P=0.022), splenectomy (HR = 0.552; 95%CI: 0.381-0.799; P=0.002) and MVI (HR=1.485, 95%CI: 1.040-2.121, P=0.029) were independent prognostic factors for overall survival (Table 4).

Therefore, patients with HCC and pathological spleen may benefit from synchronous hepatectomy and splenectomy without increased surgical risks.

### Decreased ΔNLR is related to a good prognosis of patients with HCC and pathological spleen

As reported previously, changes in systemic inflammatory response indexes may predict survival of various human cancers [10, 14, 15]. We wondered whether these indexes could predict the prognosis of patients with HCC and pathological spleen.

The changes in liver function and hypersplenism between groups A and B are present in Supplementary Table 1. After surgery, the PLT count in group A was dramatically increased compared to group B (pre-operative: 53.48±35.63 vs. 75.34±18.69, P<0.001; post-operative at 1 month: 215.45±99.37 vs. 96.48±40.74, P<0.001). However, the TBIL, AST and ALB levels were comparable the two groups.

Furthermore, inflammation-based prognostic indexes, including pre- and post-absolute neutrophil counts, the change in absolute neutrophil counts(ΔN), pre- and post-absolute lymphocyte counts, the change in absolute lymphocyte count (ΔL), pre- and post-NLR, Δ NLR, pre- and post-PLR, and Δ PLR, were made a compared between the two groups (Supplementary Table 2). Most of inflammation-based markers were significantly different between group A and group B, especially pre- lymphocyte counts (P<0.001), post-absolute lymphocyte counts (P<0.001), ΔL (P<0.001), Δ NLR (P<0.001), post-PLR (P<0.001), and Δ PLR(P<0.001).

Cox proportional hazard model analysis revealed that Δ NLR (HR=1.838, 95%CI: 1.255-2.692, P=0.002) and MVI (HR=1.813, 95%CI: 1.181-2.784, P=0.007) were significant independent prognostic factors in the univariate and multivariate analyses for overall survival among patients with HCC and pathological spleen (Table 5). Similarly, ΔNLR (HR=1.586, 95%CI: 1.167-2.156, P=0.003) and MVI (HR=1.611, 95%CI: 1.131-2.293, P=0.008) were also significant independent prognostic factors for disease-free survival for patients among groups A and B (Table 6). Thus, a decreased Δ NLR could predict a good prognosis for patients with HCC and pathological spleen.

**Table 5:** Univariate and multivariate analyses of prognostic factors for overall survival in patients with HCC and hypersplenism when systemic inflammatory response indexes were included (n=381)

Variables	Univariate analysis		Multivariate analysis	
	HR (95%CI)	P value	HR (95%CI)	P value
Gender, (F vs. M)	1.215(0.647-2.284)	0.544		
Age, (≥60 vs. <60 y)	0.926(.532-1.612)	0.786		
HBsAg, (positive vs. negative)	0.715(0.282-1.812)	0.479		
AFP, (≥400 vs.<400 ng/mL	1.241(0.789-1.951)	0.350		
TBIL	1.020(0.990-1.051)	0.189		
AST	0.996(0.986-1.006)	0.451		
ALB	0.995(0.955-1.037)	0.819		
Type of resection (major vs. minor)	0.825(0.444-1.534)	0.544		
Transfusion (yes vs. no)	0.958(0.508-1.809)	0.896		
Splenectomy, (yes vs. no)	0.772(0.254-2.343)	0.648		
Pre-PLT	0.990(0.976-1.006)	0.217		
Pre-WBC	0.898(0.562-1.435)	0.654		
Pre-N	1.783(0.896-3.661)	0.115		
Pre-L	0.435(0.162-1.168)	0.099		
Pre-NLR	0.833(0.641-1.082)	0.171		
Pre-PLR	1.004(0.992-1.015)	0.524		
Post-N	0.997(0.740-1.344)	0.986		
Post-L	1.134(0.781-1.644)	0.509		
Post-NLR	0.811(0.590-1.114)	0.196		
Post-PLR	1.000(0.991-1.008)	0.915		
ΔPLT	0.748(0.436-1.258)	0.293		
ΔN	0.584(0.323-1.057)	0.076		
ΔL	1.153(0.618-2.150)	0.655		
ΔNLR	4.000(2.180-7.340)	<0.001	1.838(1.255-2.692)	0.002
ΔPLR	0.696(0.247-1.960)	0.492		
Differentiation (W, M, L)	0.903(0.612-1.332)	0.608		
Tumor number (single vs. multiple)	1.503(0.679-3.326)	0.315		
Tumor size, (3- 5 vs. <3cm)	1.023(0.653-1.603)	0.921		
MVI (yes vs. no)	2.387(1.443-3.950)	0.001	1.813(1.181-2.784)	0.007
Cirrhosis (yes vs. no)	0.763(0.311-1.871)	0.554		

Pre-: preoperative; Post-: postoperative

**Table 6:** Univariate and multivariate analyses of prognostic factors for disease-free survival in patients with HCC and hypersplenism when systemic inflammatory response indexes were included (n=381)

Variables	Univariate analysis		Multivariate analysis	
	HR (95%CI)	P value	HR (95%CI)	P value
Gender, (F vs. M)	1.296(0.790-2.125)	0.305		
Age, (≥60 vs. <60 y)	0.767(0.503-1.171)	0.219		
HBsAg, (positive vs. negative)	0.597(0.313-1.138)	0.117		
AFP, (≥400 vs.<400 ng/mL	1.328(0.934-1.889)	0.114		
TBIL	1.015(0.991-1.040)	0.212		
AST	1.001(0.994-1.007)	0.837		
ALB	1.003(0.971-1.036)	0.847		
Type of resection (major vs. minor)	1.298(0.823-2.047)	0.262		
Transfusion (yes vs. no)	1.064(0.643-1.762)	0.809		
Splenectomy, (yes vs. no)	0.939(0.384-2.293)	0.889		
Pre-PLT	0.996(0.985-1.008)	0.510		
Pre-WBC	0.943(0.593-1.499)	0.804		
Pre-N	1.293(0.710-2.352)	0.401		
Pre-L	0.721(0.334-1.577)	0.405		
Pre-NLR	0.910(0.764-1.085)	0.294		
Pre-PLR	1.003(0.995-1.012)	0.480		
Post-N	1.027(0.846-1.246)	0.789		
Post-L	0.889(0.633-1.249)	0.497		
Post-NLR	0.857(0.697-1.054)	0.144		
Post-PLR	0.997(0.989-1.004)	0.391		
ΔPLT	0.749(0.493-1.138)	0.176		
ΔN	0.771(0.489-1.216)	0.263		
ΔL	0.904(0.561-1.459)	0.680		
ΔNLR	1.897(1.202-2.995)	0.006	1.586(1.167-2.156)	0.003
ΔPLR	0.973(0.431-2.196)	0.948		
Differentiation (W, M, L)	1.112(0.829-1.493)	0.479		
Tumor number (single vs. multiple)	0.875(0.445-1.721)	0.700		
Tumor size, (3- 5 vs. <3cm)	1.006(0.711-1.423)	0.973		
MVI (yes vs. no)	1.520(1.026-2.253)	0.037	1.611(1.131-2.293)	0.008
Cirrhosis (yes vs. no)	0.704(0.366-1.352)	0.291		

Pre-: preoperative; Post-: postoperative

## DISCUSSION

Portal hypertension is considered a surgical contraindication for HCC due to the poor prognosis and increased surgical complications [16–18]. The survival of patients with portal hypertension and/or multifocal disease is less than 30% at 5 years, regardless of Child-Pugh stage [17, 18]. Liver transplantation may be a preferred option for patients with advanced liver disease [4]. However, a shortage of donors has precluded the wide expansion of transplantation for all early HCCs associated with portal hypertension. Recently, splenectomy has been adopted as another option for HCC treatment and cirrhotic patients with no potential donor for liver transplantation [5, 6, 8]. Chen et al. identified that reported that synchronous hepatectomy and splenectomy were associated with improved 5-year tumor-free survival in patients with HCC and hypersplenism [5]. Nomura and associates concluded that splenectomy may improve liver fibrosis and result in beneficial immunological changes in cirrhotic patients with hepatitis. Improvements in antitumor mechanisms can also be expected [8]. We also conducted a case-control study and found that synchronous hepatectomy and splenectomy potentially improves disease-free survival rates and alleviates hypersplenism without increasing surgical risks for patients with HCC and hypersplenism [7]. However, the sample sizes of those studies were relatively small, and the patient populations were heterogeneous. Thus, we designed the present study with an approximately ten-year follow-up to further examine the outcomes of patients with HCC (within Milan Criteria) and pathological spleen after synchronous hepatectomy and splenectomy.

The spleen plays an important role in the immune response; however, the functional aspects of the spleen in cirrhotic patients with pathological spleen (splenomegaly and hypersplenism) are largely unknown [19, 20]. Jasnis et al. reported that splenic immune function decreases with the development of HCC [21]. A large number of activated macrophages accumulated in the spleens of tumor-bearing hosts, leading to an abnormal T cell receptorCD3 complex and suppression the immune function of T cells [22]. Ugel and associates demonstrated that the spleen is fundamentally important for tumor-induced tolerance. Splenic CD11b^+^Gr-1^int^Ly6^Chi^ cells expand in the marginal zone of the spleen, where these cells alter the normal tissue cytoarchitecture. In addition, these cells are closely associated with memory CD8+ T cells and cross-presenting tumor antigens and cause tolerization. As shown in our study, both the overall survival (P=0.023) and disease-free survival (P=0.020) in group B (with pathological spleen) were significantly worse compared to group C (without pathological spleen). A pathological spleen resulted in a longer hospital stay and more blood transfusions. Moreover, a pathological spleen was a significant risk factor for tumor recurrence and long-term survival. Thus, a pathological spleen influences the outcome of HCC patients after surgical resection.

Synchronous hepatectomy and splenectomy may be another preferred choice for patients with HCC and pathological spleen after refining the selection criteria. First, splenectomy could immediately improve low WBC and PLT counts, and reduce portal vein pressure [1, 23, 24]. Increasing PLT counts after splenectomy may potentially reduce intraoperative bleeding and surgical risk [25]. Second, splenectomy may restore the immune response to tumor progression. Shimada et al [24] and Karakantza et al [26] reported that splenectomy increased the number of natural killer (NK) cells. The modulation of CD4+ and CD8+ T cells after splenectomy plays a critical role in the immune response against cancer for [5, 8, 27]. In addition, splenectomy may significantly improve liver regeneration and ameliorate liver cirrhosis given reduction in transforming growth factor (TGF)-β1, which is produced and secreted by the spleen [28–30]. We carefully performed synchronous hepatectomy and splenectomy for small HCC and pathological spleen among patients with good preserved liver function. After long-term follow-up, we found that splenectomy prolonged the mean time to recurrence (36.26±25.77 months. vs. 25.38±22.45 months, P<0.001) and given increased long-term survival (disease-free survival, P=0.004; overall survival, P=0.025). Splenectomy was an independent prognostic factor of disease-free survival for patients with HCC and pathological spleen (P=0.002), and this finding was consistent with previous studies. Although splenectomy is associated with the occurrence of overwhelming sepsis from encapsulated microorganisms, [31, 32] our data show that the incidence of complications after splenectomy is negligible. Therefore, we suggest that synchronous hepatectomy and splenectomy should be performed for patients with HCC and pathological spleen after careful candidate selection.

Systemic inflammatory response indexes, such as NLR and PLR, which have the advantage of being readily available from routine tests of blood cell counts, could predict the prognosis of HCC patients after surgery [9, 10, 33–35]. Either an increased preoperative NLR [33, 34] or postoperative NLR [35] negatively affects the outcomes of HCC patients. However, based on the present study, we found regardless of overall or disease-free survival, only changes in NLR (increased ΔNLR) and MVI were significant independent prognostic factors for patients with HCC and pathological spleen. This result suggested that the balance between the inflammatory response and immune response may change after synchronous hepatectomy and splenectomy, and that this change may lead to a different prognosis between groups A and B.

Interestingly, splenectomy was not an independent prognostic factor when systemic inflammatory response indexes were included in analysis of the prognostic factors used to predict the outcomes of HCC and pathological spleen (Table 5 and Table 6). This result may be attrbituted to the confounding effect between splenectomy and systemic inflammatory response indexes. Indeed, splenectomy resulted in a dramatic increase in WBC, lymphocyte, and PLT counts (Supplementary Tables 1 and 2). The mean preoperative NLR was increased and the mean postoperative NLR was reduced in group A compared to group B. In addition, the percent of patients with a decreased Δ NLR in group A was significantly higher than that in group B (100/110, 90.9% vs. 135/271, 49.8%, P<0.001, Supplementary Table 2). This finding may suggest that splenectomy may result in reduction of the NLR. However, the potential mechanisms need to be further explored.

Thus, we concluded that a decreased ΔNLR could predict a good prognosis in patients with HCC and pathological spleen. Although MVI was present at the time of tumor resection, the prognosis of patients with HCC and pathological spleen may be altered due to the reduced ΔNLR after synchronous hepatectomy and splenectomy, and this information may be helpful for decision-making.

This study had several limitations. First, given that it was not a randomized study, selection bias may have occurred. Second, this study was conducted at a single center. Therefore, a large multicenter and randomized controlled study is needed to confirm the role of synchronous hepatectomy and splenectomy for patients with HCC and hypersplenism

## CONCLUSIONS

Pathological spleen influences the outcome of HCC patients. Synchronous hepatectomy and splenectomy should be a treatment option for patients with HCC and pathological spleen. The ΔNLR can predict the prognosis of those patients.

## MATERIALS AND METHODS

### Patients

**Figure 2:**
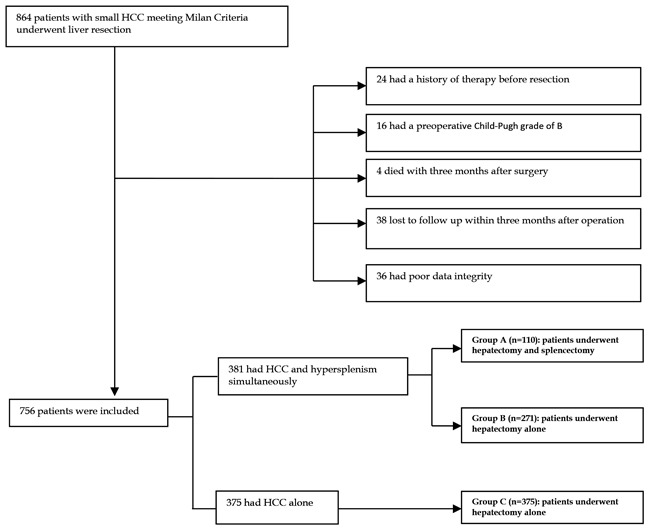
Flowchart of the process for patients’ selection

In total, 864 sequential patients with newly diagnosed HCC with Milan criteria (i.e. a single tumor < 5 cm or up to three nodules < 3 cm) who were treated at the Department of Liver Surgery and Liver Transplantation Center of the West China Hospital of Sichuan University, between February 2007 and December 2015 were prospectively enrolled. All medical records from our prospectively maintained database were reviewed retrospectively. Among the patients, 24 had received previous therapy (including radiofrequency ablation [RFA] or transhepatic arterial chemotherapy and embolization [TACE]), 16 were Child–Pugh grade B, 4 died with three months after surgery, and 38 were lost to follow-up within the first 3 months after liver resection, and data for 36 patients were of poor quality. All of these patients were excluded. Ultimately, 756 patients were included in this retrospective analysis. In total, 381 patients suffered from HCC and pathological spleen concurrently. One hundred ten of 381 patients who underwent synchronous hepatectomy and splenectomy were included in group A. The remaining 271 patients who received a hepatectomy alone were included in group B. Finally, 375 HCC patients without pathological spleen who underwent hepatectomy alone were included in group C. Details about patient selection are presented in Figure 2. In addition, the diagnosis of HCC was confirmed by a postoperative histopathologic examination. Clinical variables, including demographic data, complete blood counts differentiation assessments, PLT, WBC, splenomegaly, liver function tests, AFP, HBV markers, and staging of the tumor (including the number of focal hepatic lesions, maximum diameter detected, degree of differentiation, micro-vascular invasion(MVI), and Ishak score), were collected.

The following data were also analyzed for each group: length of hospital stay, follow-up time, major complications (Dindo et al [36]. classification of surgical complications), type of liver resection (major vs. minor), intraoperative bleeding and number of transfusions.

This study complied with the standards of the Helsinki Declaration and current ethical guidelines and was approved by the Ethics Committee of West China Hospital, Sichuan University.

### Indications for synchronous hepatectomy and splenectomy

A platelet count less than 100 × 10^9^/l and/or a WBC count less than 3.0 × 10^9^/l were defined as hypersplenism. Splenomegaly and hypersplenism were defined as a pathological spleen.

As previously described,[5, 7] patients who underwent synchronous splenectomy exhibited either splenomegaly classified as grade I or higher (spleen enlargement beyond the left subcostal margin and palpable) with a concurrent WBC count of less than 3.0 × 10^9^/l and PLT count less than 80 × 10^9^/l; or grade I or greater splenomegaly with a WBC count of less than 2.0 × 10^9^/l or a PLT count below 50 × 10^9^/l.

Indications for hepatectomy included normal liver function without minor fluid retention requiring diuretic therapy, no history of variceal bleeding, and no evidence of extrahepatic venous invasion or lymph node or distant metastases.

### Definition and calculation of systemic inflammatory response indexes

All preoperative WBC counts and differential counts were obtained 2 days before the operation.

The NLR was calculated from the differential count by dividing the absolute neutrophil count (N) by the absolute lymphocyte count (L). The postoperative NLR was obtained at the first follow-up visit at the outpatient department 1 month after the operation. ΔNLR was calculated by subtracting the preoperative NLR from postoperative NLR. If the NLR value was ≤0, ΔNLR was defined as decreased. Otherwise, it was defined as increased.

The PLR was calculated from the differential count by dividing the absolute PLT count by the absolute lymphocyte count. The postoperative PLR was obtained at the same time as the NLR. The ΔPLR was calculated by subtracting the preoperative PLR from the postoperative PLR. If the PLR value was ≤0, the ΔPLR was defined as decreased. Otherwise, it was defined as increased.

### Follow-up

All of the patients received follow-up monitoring 1 mo after the operation, every 3 mo thereafter during the first 3 years and then every 6 mo in subsequent years.

Physical examination, blood cell and differential counts, liver function tests, AFP levels, HBV markers and HBV-DNA levels (if the patient was diagnosed with HBV infection), and imaging examinations were included when necessary in the follow-up examinations. Tumor recurrence was diagnosed based on the identification of a new lesion in at least two radiological examinations and increased AFP levels. OS time was defined as the interval between the operation and death or the last follow-up. Disease-free survival (DFS) time was defined as the time interval between the operation and the 1st incidence of detectable recurrence. The last follow-up date was the end of July 2016.

### Statistical analysis

Continuous variables are expressed as mean ± standard deviation and were compared between groups using the *t* test or Mann Whitney *U* test for variables with an abnormal distribution. Categorical data were compared using the χ^2^ test or Fisher's exact test. The overall survival rates were analyzed using the Kaplan Meier method, and the differences were analyzed using the log-rank test. The Cox proportional hazard model was used for univariate and multivariate analyses of prognostic factors after surgery. Two-tailed *P* values ≤ 0.05 were considered statistically significant. Calculations were performed using the SPSS package (SPSS, Inc, Chicago, IL).

## SUPPLEMENTARY FIGURES AND TABLES


