# Supplementary material for: Synchronous splenectomy and hepatectomy for patients with small hepatocellular carcinoma and pathological spleen: neutrophil to lymphocyte ratio changes can predict the prognosis
Source: Oncotarget. 2017 May 10;8(28):46298–311. doi: 10.18632/oncotarget.17758 (PMC5542268; doi:10.18632/oncotarget.17758)
Supplement: Supplementary file 1 [file oncotarget-08-46298-s001.pdf]

Synchronous splenectomy and hepatectomy for patients with small hepatocellular carcinoma and pathological spleen: neutrophil to lymphocyte ratio changes can predict the prognosis

SUPPLEMENTARY MATERIALS

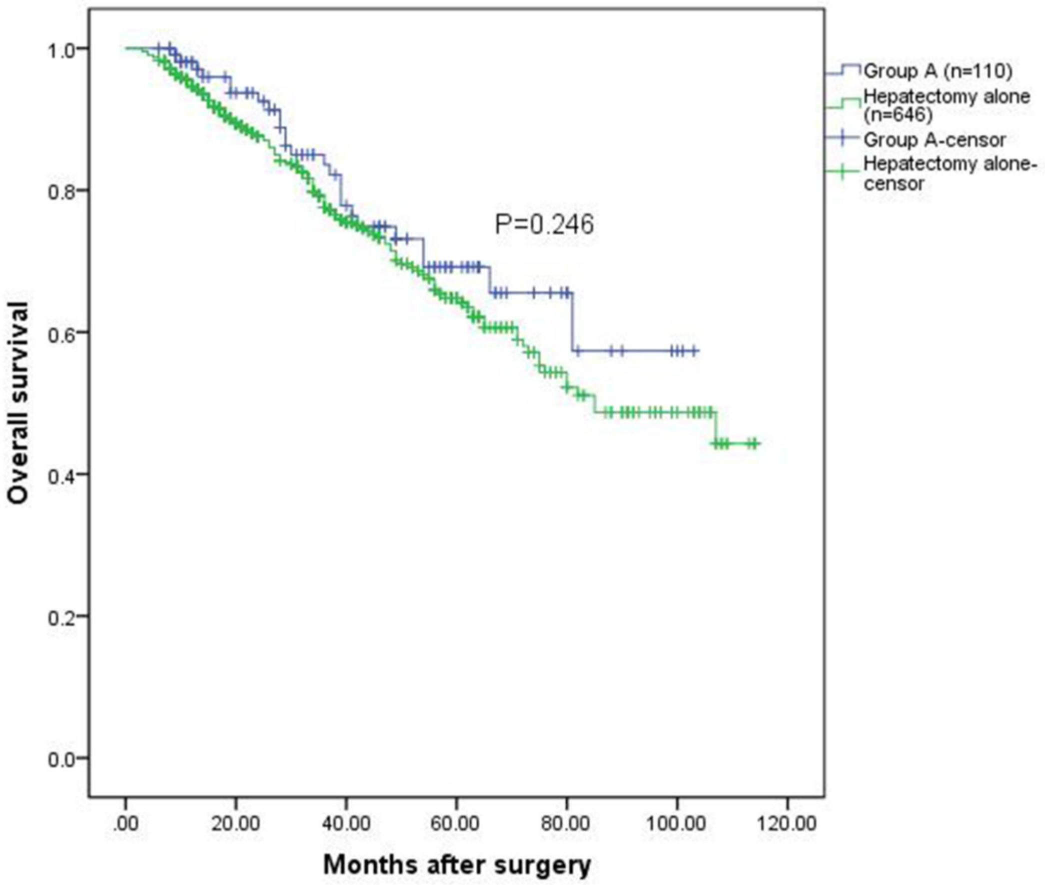

Supplementary Figure 1A: Overall survival rates for the patients in the group A and the hepatectomy alone group. The 1-, 3-, 5-, 7-, 9-year overall survival for the patients in the group A were 98.1%, 83.6%, 69.2%, 57.3%, 57.3%, respectively. And 1-, 3-, 5-, 7-, 9-year overall survival for the patients in the hepatectomy alone group were 94.7%, 77.6%, 64.8%, 51.1%, 44.3%, respectively (P=0.246).

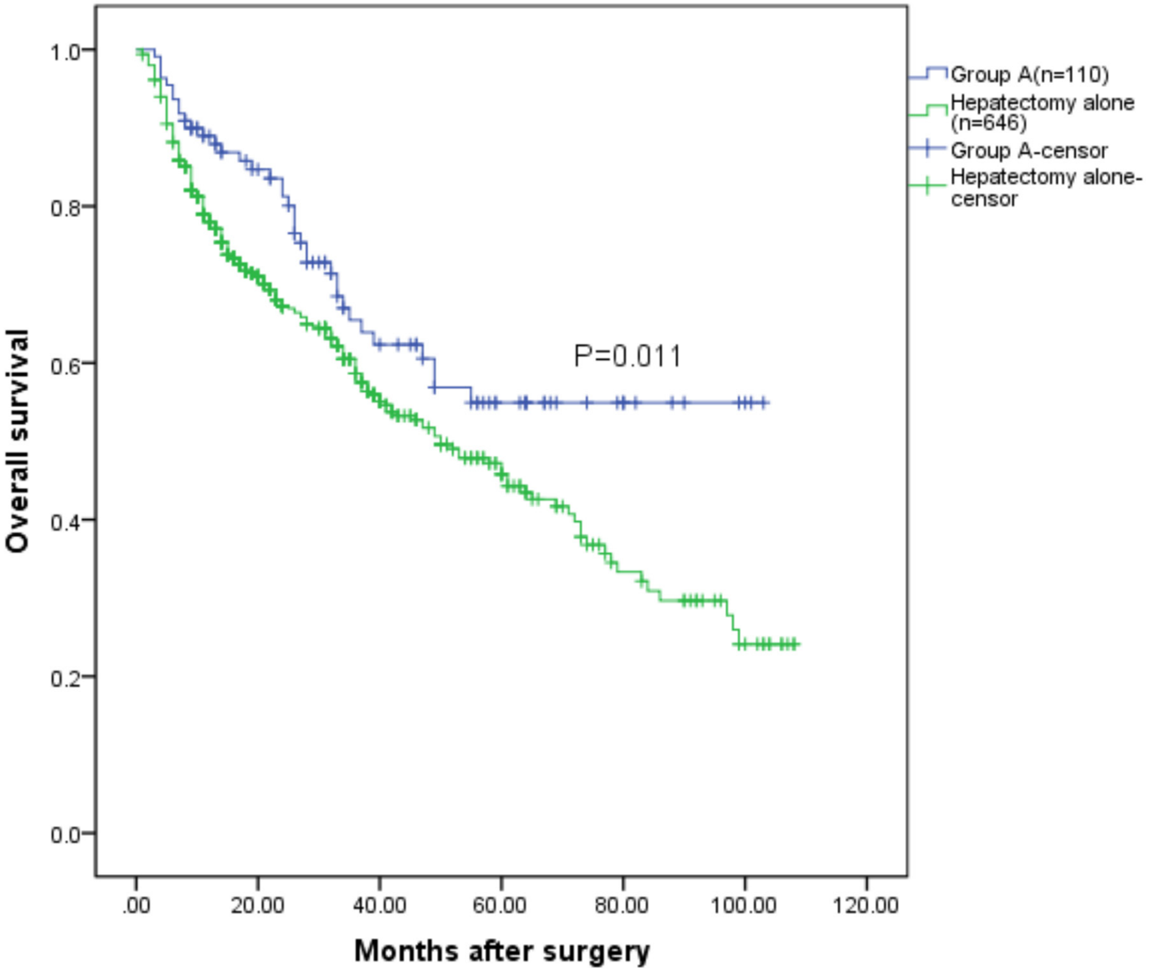

Supplementary Figure 1B: Disease-free survival rates for the patients in the group A and the hepatectomy alone group. The 1-, 3-, 5-, 7-, 9-year disease-free survival for the patients in the group A were 89.0%, 65.5%, 54.9%, 54.9%, 54.9%, respectively. And 1-, 3-, 5-, 7-, 9-year disease-free survival for the patients in the hepatectomy alone group were 78.0%, 58.7%, 45.8%, 30.9%, 24.1%, respectively (P<0.001).

Supplementary Table 1: The changes of laboratory tests about liver function, PLT and WBC before and after surgery

Variables	Pre-operation			one month after surgery		
	Group A	Group B	P	Group A	Group B	P
TBIL (μmol/L)	17.84±6.66	16.61±6.78	0.586	16.51±6.99	18.35±10.86	0.279
AST(IU/L)	40.85±19.95	43.13±26.21	0.206	53.22±35.30	50.21±83.68	0.514
ALB(g/L)	39.94±6.04	41.54±4.64	0.139	39.06±4.46	40.78±5.10	0.324
PLT(10^9/L)	53.48±35.63	75.34±18.69	<0.001	215.45±99.37	96.48±40.74	<0.001
WBC (10^9/L)	3.01±1.48	4.53±1.66	0.286	6.48±2.05	5.48±2.37	0.099

Supplementary Table 2: The changes of inflammation-based prognostic indexes between two groups before and after surgery

Variables	Group A	Group B	P
Pre-PLT (10^9/L)	53.48±35.63	75.34±18.69	<0.001
Post-PLT (10^9/L)	215.45±99.37	96.48±40.74	<0.001
ΔPLT			
Mean	160.82±97.99	21.14±36.53	<0.001
Increase, n (%)	108(98.2%)	200(73.8%)	<0.001
Decrease, n (%)	2(1.8%)	71(26.2%)	
Pre-absolute neutrophil counts (10^9/L)	1.97±1.26	2.69±1.32	0.806
Post-absolute neutrophil counts (10^9/L)	2.90±1.32	3.47±2.15	0.026
ΔN (10^9/L)			
Mean	0.93±1.67	0.79±2.30	0.304
Increase, n (%)	88(80.0%)	183(67.5%)	0.018
Decrease, n (%)	22(20.0%)	88(32.5%)	
Pre-absolute lymphocyte counts (10^9/L)	0.74±0.31	1.32±0.57	<0.001
Post-absolute lymphocyte counts (10^9/L)	2.84±1.23	1.44±0.59	<0.001
ΔL (10^9/L)			
Mean	2.10±1.23	0.13±0.60	<0.001
Increase, n (%)	110(100.0%)	155(57.1%)	<0.001
Decrease, n (%)	0(0.0%)	116(42.9%)	
Pre-NLR	3.21±3.63	2.40±2.09	0.004
Post-NLR	1.24±0.91	3.32±8.74	0.047
Δ NLR			
Mean	-1.96±3.70	0.93±8.76	0.529
Increase, n (%)	10(9.1%)	136(50.2%)	<0.001
Decrease, n (%)	100(90.9%)	135(49.8%)	
Pre-PLR	81.71±61.82	69.36±40.30	0.015
Post-PLR	89.83±62.81	19.18±8.62	<0.001
Δ PLR			
Mean	5.83±84.07	-50.18±39.48	<0.001
Increase, n (%)	58(52.7%)	1(0.4%)	<0.001
Decrease, n (%)	52(47.3%)	270(99.6%)	
